# An Immunoregulatory Role of Interleukin-3 in Allergic Asthma

**DOI:** 10.3389/fimmu.2022.821658

**Published:** 2022-02-23

**Authors:** Susanne Krammer, Zuqin Yang, Theodor Zimmermann, Paraskevi Xepapadaki, Carol I. Geppert, Nikolaos G. Papadopoulos, Susetta Finotto

**Affiliations:** ^1^ Department of Molecular Pneumology, Friedrich-Alexander-University (FAU) Erlangen-Nürnberg, Universitätsklinikum Erlangen, Erlangen, Germany; ^2^ Children’s Hospital, Department of Allergy and Pneumology, Friedrich-Alexander-University (FAU) Erlangen-Nürnberg, Universitätsklinikum Erlangen, Erlangen, Germany; ^3^ Allergy and Clinical Immunology Unit, 2nd Pediatric Clinic, National and Kapodistrian University of Athens, Athens, Greece; ^4^ Institute of Pathology, Friedrich-Alexander-University (FAU) Erlangen-Nürnberg, Universitätsklinikum Erlangen, Erlangen, Germany; ^5^ Centre for Respiratory Medicine & Allergy, Division of Infection, Immunity & Respiratory Medicine, University of Manchester, Manchester, United Kingdom

**Keywords:** allergic asthma, eosinophils, IL-3, ILC2s, T regulatory cells

## Abstract

**Background:**

Allergic asthma is a chronic airway inflammatory disease associated with airway mucus hyper-production. ILC2 cells, which express the Th2 transcription factor GATA3, have been associated with allergic asthma. The cytokine IL-3 is known to support eosinophil, basophil and mucosal mast cell differentiation and survival; however, its role on T regulatory cells as well as on lung ILC2 and in pediatric asthma needs further investigation.

**Objectives:**

To investigate the role of IL-3 in preschool children and to explore its therapeutic role in experimental asthma.

**Methods:**

In a cohort of preschool children with and without asthma, we analyzed the secretion of IL-3 in nasopharyngeal fluid (NPF) and IL-3 receptor (R) alpha chain mRNA expression in peripheral blood mononuclear cells (PBMCs). In a murine model of allergic asthma, we analyzed the phenotype of wild-type untreated and rIL-3 intranasally treated asthmatic mice.

**Results:**

IL-3 was found downregulated in the nasopharyngeal fluid of children with partially controlled asthma, as compared to control children. Moreover, IL-3 was found induced in phytohemagglutinin (PHA)-stimulated PBMCs from children with asthma and treated with steroids. Finally, IL-3 in NPF directly correlated with the anti-inflammatory molecule sST2 in steroid-treated asthmatic children. Intranasal rIL-3 delivery *in vivo* during the challenge phase decreased airway mucus production and inflammatory eosinophils. Moreover, rIL-3 given during the challenge phase, reduced lung ST2^int^GATA3+ILC2, accompanied by an induction of T regulatory cells in the airways.

**Conclusions:**

IL-3 was found associated with steroid-resolved asthma. Moreover, treatment with rIL-3 resulted in amelioration of airway eosinophilia and mucus production, two main pathophysiological conditions associated with asthma in a murine model of allergic asthma. Thus, rIL-3 opens new strategies for immunotherapy of this disease.

## Introduction

Allergic asthma (AA) is a chronic inflammatory disease of the airways affecting especially children worldwide. The immunopathology of this disease has been associated with the damage induced to the airways by repeated respiratory triggers like allergens, viruses and bacterial infections in a distinct genetic background. The respiratory epithelium represents the first defense barrier of the organism to the external insults (1). The epithelium signals downstream with molecules known as alarmins, which induce the development and recruitment of innate lymphoid cell type 2 (ILC2) (2–6), which initiate a cascade of cellular events resulting in the pathological expansion of a TH2 type immune response to otherwise innocuous airborne allergens (7). However, the events associated with the resolution of AA during acute episodes are yet to be defined. It has been suggested that, allergen-inducible CD4+CD25+Foxp-3+ T regulatory cells limit inflammation and maintain immune homeostasis in the lung. These cells are induced by TGF-beta and release IL-10 (8, 9). The exact mechanism by which, upon allergen challenge or infection, T cells are activated and then drive the airway inflammation seen in allergic asthma needs to be defined (10–12). This airway inflammation encloses the recruitment of cells of the innate immunity, which clear the antigen or infection and finally return to a homeostatic T-immunosuppressive status.

IL-3 promotes the differentiation of multipotent hematopoietic stem cells into myeloid progenitor cells or, with the addition of IL-7, into lymphoid progenitor cells. IL-3 is secreted by basophils and activated T cells. It supports the growth and differentiation of mucosal mast cells as well as T cells from the bone marrow (13). Moreover, although IL-3 was found in the bronchial airways of both asthmatic and control subjects after the allergen challenge, its increase did not correlate with airway hyperresponsiveness, metacromasia or eosinophil activation in allergen-induced asthma (14). Thus, the role of IL-3 in asthma needs further investigation. As mentioned above, another type of infiltrating cells into the site of inflammation in the lung of asthmatic patients is ILC2. Although these cells do not express lineage markers like CD3 or CD4, they show effector functions like Th2 cells expressing IL-5 and IL-13, as well as GATA-binding protein 3 (GATA3) (15–17). ILC2s are developed from common lymphoid precursors (CLP) in the bone marrow upon stimulation mainly with IL-25, IL-33 and thymic stromal lymphopoietin (TLSP), which are released by damaged or allergen- or virus-activated airway epithelial cells (18, 19). In addition, recently, TGF-beta has been shown to support the development of CLP into ILC2 *via* expression of the IL-33 receptor gene *Il1rl1* (encoding IL-1 receptor-like 1, also known as ST2) in ILC2p and common helper-like innate lymphoid progenitors (CHILP) (20). Previous studies have also shown that IL-25, predominantly induces the development of inflammatory (i) ILC2s, while IL-33 preferentially increases tissue-resident natural (n) ILC2s (21, 22). Moreover, we and others have recently demonstrated that 25(OH)VitD3 enhances the production of the soluble (s) ST2 isoform. Because soluble ST2 (sST2) neutralizes the effect of IL33, sST2 is considered an anti-inflammatory factor in asthma (4, 23, 24). However, it is unknown if ILC2s are influenced by IL-3 *via* IL-3R expression, in general, and in asthma, in particular.

To investigate this hypothesis, we analyzed the secretion of IL-3 in preschool children with and without asthma, correlated its regulation in different asthma phenotypes and treatment, and studied wild-type (WT) mice treated with recombinant IL-3 in an experimental model of allergic asthma.

## Methods

### Human Study PreDicta

In the European study PreDicta (Post-infectious immune reprogramming and its association with persistence and chronicity of respiratory allergic diseases), we examined a cohort of children with and without asthma at the age of 4 to 6 years. The study was performed in collaboration with the children’s hospital in Erlangen and approved by the local ethics committee of the Friedrich-Alexander University Erlangen-Nürnberg, Germany (Re-No 4435). The study is registered in the German Clinical Trials Register (www.germanctr.de: DRKS00004914). The recruitment of the two cohorts of preschool children, the timescale for the clinical visits, the inclusion and exclusion criteria and the data collection were described recently (4, 5, 25–28) and are reported in other forms in **Table 1**, **Supplementary Tables 1** and **2** along with the relevant clinical aspects and characteristics.

**Table 1:** Clinical data of the asthma PreDicta cohort WP1-UK-ER analyzed at the baseline visit.

Patient ID	Age at B0 [years]	Gender	Asthma severity (GINA 2005)	Asthma control (GINA 2009)	Treatment	PBMC SN PHA IL3 [pg/ml]
A1	6	Male	I	Controlled	Steroid	152,182
A2	6	Male	II	Partially controlled	Steroid	75,870
A3	5	Female	II	Partially controlled	Steroid	22,678
A4	6	Male	II	Controlled	Steroid	48,344
A5	5	Male	I	Partially controlled	Steroid	25,122
A6	5	Female	I	Controlled	Steroid	51,480
A7	5	Male	I	Partially controlled	Steroid	58,026
A9	4	Female	n.a.	Partially controlled	Steroid	n.m.
A10	6	Female	I	Partially controlled	Non-steroid	n.m.
A12	5	Male	II	Controlled	Steroid	n.m.
A13	4	Male	III	Partially controlled	Steroid	38,544
A16	5	Female	III	Uncontrolled	Steroid	6,436
A17	6	Female	I	Uncontrolled	Steroid	0,000
A23	5	Male	I	Controlled	Steroid	14,320
A24	4	Female	I	Controlled	Steroid	64,980
A25	4	Male	I	Controlled	Steroid	0,000
A28	5	Male	I	Controlled	Non-steroid	0,000
A29	4	Male	I	Controlled	Non-steroid	5,082
A30	5	Male	I	Controlled	Non-steroid	0,000
A31	4	Male	I	Partially controlled	Steroid	0,000
A38	4	Male	I	Controlled	Steroid	50,610
A39	5	Female	I	Controlled	Non-steroid	0,000
A42	5	Male	II	Controlled	Steroid	0,000
A43	5	Female	II	Uncontrolled	Steroid	0,000

Asthma severity: I = intermittent: FEV1 > 80%; MEF > 65%; symptom-free interval > 2 months. II = mild persistent: FEV1 > 80%; MEF > 65%; symptom-free Interval < 2 months. III = moderate persistent: FEV1 < 80%; MEF < 65%; symptoms several days a week. IV = severe persistent: FEV1 < 60%; symptoms during the day and night.

FEV1, forced expiratory volume in 1 s; MEF, maximal expiratory flow; PBMC, peripheral blood mononuclear cell; PHA, phytohemagglutinin.

Further, blood was collected for the isolation and culture of peripheral blood mononuclear cells (PBMCs) and subsequent further analysis.

### Isolation of Human Peripheral Blood Mononuclear Cells and Cell Culture

Heparinized blood was collected from children with and without asthma at the Baseline Visit of the PreDicta study. Immediately after, PBMCs were isolated with Ficoll using density centrifugation. Afterward, one part of the PBMCs was used for extraction of total mRNA by using QIAzol Lysis Reagent (Qiagen, Hilden, Germany), according to the manufacturer’s protocol and subsequent gene expression analysis, as described below. The remaining cells were cultured at a concentration of 1 × 10^6^ cells/ml for 48 h in Roswell Park Memorial Institute (RPMI) 1640 medium supplemented with 25 mmol/L of HEPES, 100 IU/ml of penicillin, 100 µg/ml of streptomycin, 1% l-glutamine (200 mmol/L) (all from Anprotec, Bruckberg, Germany), 50 µmol/L of β-mercaptoethanol, 1% MEM Vitamin (all from Sigma-Aldrich, Steinheim, Germany), 1% non-essential amino acids, 1% sodium pyruvate (all from Gibco^®^, Thermo Fisher Scientific, Waltham, MA, USA), and 10% fetal bovine serum (FBS) (Biochrom GmbH, Berlin, Germany) at 37°C and 5% CO_2_ and stimulated with 10 µg/ml phytohemagglutinin (PHA) (Sigma-Aldrich, Steinheim, Germany).

Human IL-3 was detected in the cell-culture supernatants by using the IL-3 DuoSet ELISA kit from R&D (Wiesbaden, Germany) according to the manufacturer’s protocol.

### Nasopharyngeal Fluid Collection With Swab

For the detection of IL-3 in the upper airways of the children, a nasopharyngeal specimen was collected using a per-nasal applicator swab with a flocked soft nylon fiber tip (E-Swab 482CE; Copan, Brescia, Italy) as described in detail elsewhere (2). ELISA from R&D Systems (Minneapolis, MN, USA) was used to detect IL-3 (DY203) and sST2 (DY523B-05) in human nasopharyngeal fluid (NPF).

### Mice

All mice used in this study have Balb/c genetic background. The Balb/c WT mice were obtained from Janvier Labs (Saint-Berthevin, France). All mice were maintained under specific pathogen-free conditions and had free access to food and water. All experiments were performed in accordance with the German and European laws for animal protection and were approved by the government of Unterfranken, Bavaria (Az. 54-2532.1-2/10).

### Ovalbumin Sensitization and Challenge

For the induction of ovalbumin (OVA)-dependent allergic asthma, WT female mice at the age of 6–8 weeks were sensitized twice at days 0 and 7 with intraperitoneal (i.p.) injections of 100 µg of OVA (Calbiochem, San Diego, CA, USA) complexed with 10% aluminum potassium sulfate (Sigma Aldrich, Steinheim, Germany). Thereafter, on days 18, 19, and 20, the mice were challenged intranasally (i.n.) with 50 µg of OVA, dissolved in phosphate-buffered saline (PBS). On day 21, the animals were sacrificed to measure the invasive lung function, obtain bronchoalveolar lavage (BAL) and isolate and analyze total lung cells.

### rIL-3 Treatment *In Vivo*

Mice were anesthetized with isoflurane and given 5 µg of murine rIL-3 (PeproTech, Hamburg, Germany) i.n. at different time points. This procedure was done during the sensitization phase (days 0 and 17) in the rIL-3 sensitization group and during the challenge phase on days 19 and 20 in the rIL-3 challenge group. If OVA was applied on the same day; the IL-3 treatment was done 30 min before OVA i.p. injection or 1 h before OVA i.n. application.

### Analysis of Airway Hyperresponsiveness

The invasive lung function of the mice was measured by the Flexivent FX System (Scireq, Emka, Montreal, QC, Canada) on day 21. The mice were anesthetized, and a tracheotomy with a 16-G cannula was performed. We challenged the mice with increasing doses (0, 5, 10, 25, and 50 mg/ml) of nebulized methacholine (MCh) to obtain the respiratory resistance (Rrs) values.

### Histology

Lungs were removed, then fixed in 4% formaldehyde, dehydrated, and embedded in paraffin. Serial paraffin sections (3 µm) were stained with H&E for semiquantification of inflammation. The scoring of the perivascular and peribronchial inflammation was gathered blindly by a pathologist as previously described in detail (29). For the quantification of mucus production, lung sections were prepared as described above and stained with periodic acid-Schiff (PAS). For the analysis, the PAS score was determined *via* semiquantified classification of bronchi regarding the size of PAS-positive areas.

### Analysis of the Bronchoalveolar Lavage Cells

BAL was obtained on day 21 by intratracheal injection and aspiration of 0.8 ml of Sterofundin (B. Braun Melsungen AG, Melsungen, Germany) twice. BAL cells were harvested for flow cytometry analysis after centrifugation for 5 min at 1.500 rpm. Anti-CD45.2, anti-siglec F, anti-CD62L, anti-CD101, and anti-CD125 were used for the staining of eosinophils in BAL. The staining procedure of the BAL was done in the same way as the lung cell flow cytometry staining.

### Total Lung Cell Isolation

Lungs were removed on day 21 of the asthma protocol, and total cells were isolated as previously described (30). Briefly, lung tissue was cut into small pieces by a scalpel and digested with 300 U/ml of Collagenase Type Ia and 0.015% DNase (10 mg/ml) in PBS at 37°C for 1 h. Digested lung was then strained through a 40-µm cell strainer and subsequently lysed with an Ammonium-Chloride-Potassium (ACK)-Lysis buffer (0.15 M of NH_4_Cl, 0.1 mM of KHCO_3_, and 0.1 mM of Na_2_-EDTA dissolved in ddH_2_O). Finally, the lung cells were washed with PBS and counted using a hemocytometer. Isolated cells were plated at a concentration of 1 × 10^6^ cells/ml in a 48-well plate and cultured in RPMI 1640 medium containing 100 IU/ml of penicillin, 100 µg/ml of streptomycin (all from Anprotec, Bruckberg, Germany), and 10% fetal calf serum (FCS) (Biochrom GmbH, Berlin, Germany) at 37°C and 5% CO_2_ for 24 h. After 24 h, cell culture supernatants were harvested and analyzed by quantitative ELISA.

### Lung ILC2 Skewing

To expand ILC2s *in vitro*, 1 million lung cells in 1 ml of RPMI 1640 medium containing 100 IU/ml of penicillin, 100 µg/ml of streptomycin (all from Anprotec, Bruckberg, Germany), and 10% FCS (Biochrom GmbH, Berlin, Germany) were stimulated with 20 ng/ml of rmIL-2, 10 ng/ml of rmIL-33 (Immunotools GmbH, Friesoythe, Germany), and 10 ng/ml rmTSLP Invitrogen, Thermo fisher scientific, Waltham, MA, USA); after 5 days, the cells and cell culture supernatants were harvested and analyzed by flow cytometry and ELISA, respectively. In another experimental setting, we differentiated ILC2s as described above. Briefly, we restimulated skewed ILC2 on day 5 with 20 ng/ml of rIL-2, 10 ng/ml of rIL-33, and with or without 5 ng/ml of rIL-3 for further culture until day 12.

### Flow Cytometry Analysis (FACS) of Murine Lung Cells

Single-cell suspensions from the murine lung were incubated with the respective mix of surface antibodies diluted in fluorescence-activated cell scanning (FACS) buffer (1× PBS, 1% FCS, 0.1% NaN_3_) for 30 min in the dark at 4°C. After the cells were washed once in FACS buffer, cells were fixed in 2% paraformaldehyde (PFA) for flow cytometric analysis. For subsequent intracellular staining, cells were fixed and permeabilized with Fixation/Permeabilization buffer (eBioscience, Thermo Fisher Scientific, Waltham, MA, USA) in accordance with the manufacturer’s protocol for 35 min in the dark at 4°C followed by intracellular staining with different transcriptions markers diluted in 1× Permeabilization buffer (eBioscience, Thermo Fisher Scientific, Waltham, MA, USA) in the dark at 4°C for 30 min. Afterward, cells were washed and resuspended in FACS buffer for analysis. For ILC2 staining, a premixed lineage cocktail (Miltenyi Biotec, Bergisch Gladbach, Germany) containing biotinylated anti-CD5, anti-CD11b, anti-CD45R (B220), anti-7-4, anti-Gr-1 (Ly-6G/C) and anti-Ter-119 monoclonal antibodies were used according to the manufacturer’s protocol. Streptavidin conjugated to APC was applied in secondary staining together with the APC-conjugated surface markers anti-CD3, anti-CD4, anti-CD11c, anti-Siglec F, and further surface antibodies against anti-KLRG1, anti-Thy1.2, anti-ST2, and anti-IL3Rα followed by intracellular staining with fluorochrome-coupled anti-GATA3 as described above. For Treg staining, anti-CD4 and anti-CD25 antibodies were used for surface staining and anti-Foxp3 for intracellular staining. Flow cytometry was performed on FACS Canto II (BD Biosciences, Heidelberg, Germany), and flow cytometry data were analyzed by FlowJo v10 (TreeStar Inc., San Jose, CA, USA). All of the antibodies used for flow cytometry analysis are shown in **Supplementary Table 3**.

### Enzyme-Linked Immunosorbent Assay

Serum levels of IgE were measured using the OptEIA™ sandwich ELISA kit from BD Bioscience (Heidelberg, Germany). Mouse IL-10 and TGF-β1 from cell culture supernatants were quantified by OptEIA™ sandwich ELISA kit from BD Bioscience (Heidelberg, Germany) and DuoSet sandwich ELISA kits from R&D System (Wiesbaden, Germany), respectively. Each ELISA was performed according to the manufacturer’s instructions.

### Statistical Analysis

All statistical analyses were performed using GraphPad Prism v8 for Windows (GraphPad, La Jolla, CA, USA). All datasets were analyzed for normal distribution with the Shapiro–Wilk normality test before statistical analysis was performed. Statistical significances were calculated using a two‐tailed Student’s *t*-test for the analysis of two-group comparisons and one- or two-way ANOVA for multiple comparisons to generate p‐value data (*p ≤ 0.05, **p ≤ 0.01, ***p ≤ 0.001, ****p ≤ 0.0001) or the non-parametric equivalent. Correlations were examined by linear regression analysis. The two-tailed Pearson correlation analysis was performed to obtain r and p values. For *post-hoc* analysis, we used Tukey’s method test (one-way ANOVA) and Sidak’s method test (two-way ANOVA). Unless otherwise indicated, data are presented as mean ± SEM.

## Results

### IL-3 Is Associated With Controlled and Steroid-Treated Asthma in Preschool Children

**Figure 1:**
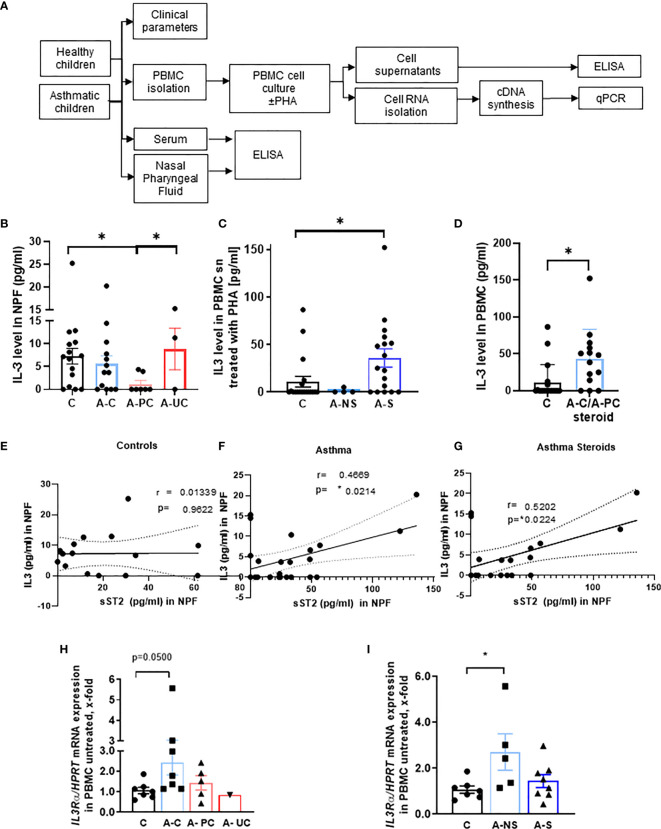
Role of IL-3 in asthmatic patients. **(A)** Experimental design of the study PreDicta on the children cohorts analyzed in this study. **(B)** ELISA analysis of IL-3 levels in the nasopharyngeal fluid (NPF) from control preschooler and asthmatic preschooler subgrouped into asthma controlled **(A–C)**, asthma partially controlled (A-PC), and asthma uncontrolled (A-UC) (GINA 2009) (n = 15, 13, 7, 3). **(C)** ELISA analysis of IL-3 levels in the supernatant from peripheral blood mononuclear cells (PBMCs) isolated from healthy control and asthmatic children and cultured with phytohemagglutinin (PHA) for 24 h. Asthmatic children were subgrouped in accordance to the medications used: non-steroids (NS) and steroids (S) (n = 19, 4, 18; p = 0.028). **(D)** Analysis (shown in panel **C**) considering steroid-treated asthmatic children controlled and partially controlled (n = 19, 14). **(E–G)** IL-3 in NPF from control **(E)** (n = 15) and asthmatic preschoolers **(F)** (n = 24) were correlated with the correspondent sST2 in NPF. A significant positive correlation was maintained when only the asthmatic children treated with steroids were correlated **(G)** (n = 19) (Pearson’s correlation test). **(H)** *IL3Ra/HPRT* mRNA expression in PBMCs of controls and asthmatic children subgrouped into asthma controlled **(A–C)**, asthma partially controlled (A-PC), and asthma uncontrolled (A-UC) (n = 7, 7, 5, 1) and **(I)** in asthmatic children treated with (A-S) or without steroids (A-NS) at the baseline visit (n = 7, 5, 8). Data are presented as means ± SEMs. Two-tailed Student’s *t*-test or Kruskal–Wallis test was used to calculate statistical significance. *p ≤ 0.05.

To start investigating the function of IL-3 in allergic asthma, we analyzed the IL-3 secretion in the NPF of the cohort of our study PreDicta including 21 healthy controls and 24 asthmatic preschool children (**Figure 1A**, **Table 1** and **Supplementary Table 1**). Here we found that, IL-3 in the NPF of control preschoolers was comparable to the level measured in asthmatic preschoolers with controlled asthma (A–C) (GINA 2009) (**Figure 1B**). By contrast, IL-3 levels in the NPF of asthmatic preschoolers with partially controlled asthma (A-PC) were significantly reduced. We also had 3 children with uncontrolled asthma who had scattered levels of IL-3 in the NPF. However, by looking closely at their clinical data (**Supplementary Table 2**), we found that the child with no IL-3 in the NPF had worse lung function and higher C-reactive protein (CRP), confirming a possible role of IL-3 in the amelioration of asthma. Because IL-3 is known to be released by activated T cells, we challenged the PBMCs with lectin PHA. Here we found an increased secretion of IL-3 in PHA-stimulated PBMCs from asthmatic children treated with steroids (**Figure 1C**) compared to healthy children. In addition, we analyzed children treated with steroids with controlled and partially controlled asthma and found that IL-3 was induced in this group (**Figure 1D**).

### IL-3 in Nasopharyngeal Fluid Correlated With the ILC2 Anti-Inflammatory Marker Soluble ST2 in Nasopharyngeal Fluid of Asthmatic Children Treated With Steroids

To analyze the link between IL-3, ILC2, and the amelioration of asthma, we reasoned that IL-3 might be involved in the regulation of the inflammatory and anti-inflammatory properties of ILC2 *via* the regulation of the anti-inflammatory form of ST2 known as sST2. Here we found that, IL-3 and sST2 directly correlated in the NPF of asthmatics but not control children (**Figures 1E, F**). Moreover, this correlation was maintained also when only steroid-treated asthmatics were considered (**Figure 1G**). In summary, we found evidence that IL-3 might be involved in the shedding of the ST2, thus neutralizing IL-33 function in the airways.

### IL3Rα Chain Expression Is Associated With Controlled Asthma in Children

We next analyzed the mRNA expression of the specific IL-3 receptor (IL3R) alpha chain in unstimulated PBMCs. Therefore, we wanted to correlate the expression of the IL3R alpha chain in untreated PBMCs and the clinical parameter of the asthmatic preschoolers and found a significant induction of IL-3R alpha chain/HPRT mRNA expression in asthmatic children with controlled asthma as well as in those treated with non-steroid medications (**Figures 1H, I**). Taken together, we found that both the IL-3 and its specific receptor alpha chain were upregulated in controlled asthma.

### Intranasal Treatment With rIL-3 During the Challenge Phase Inhibited Airway Inflammatory Eosinophils and Mucus Production in Asthmatic Mice

**Figure 2:**
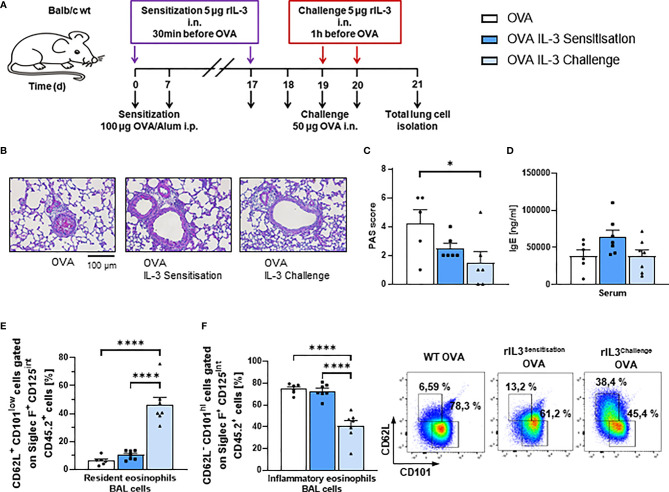
Intranasal treatment with rIL-3 during the challenge phase inhibited airway eosinophil inflammation and mucus production in asthmatic mice. **(A)** Experimental design for the induction of allergic asthma in wild-type (WT) mice treated with rIL-3 during sensitization or challenge phase for the analysis of lung ILC2s. **(B, C)** Analysis of the mucus production in the airways. A representative light microscopic picture of the periodic acid-Schiff (PAS) staining of lung sections is shown for each group (n = 6/6/6). **(D)** Serum IgE level of WT mice with asthma and with and without additional rIL-3 treatment measured by ELISA (n = 6/7/7). **(E, F)** Flow cytometry analysis of resident (CD62L+CD101^low^) and inflammatory (CD62L−CD101^high^) eosinophils in the bronchoalveolar lavage (BAL) cells. A representative dot plot is shown for each group (n = 6/7/7). Data are presented as means ± SEMs. One-way ANOVA or Kruskal–Wallis test was used to calculate statistical significance. *p ≤ 0.05; ****p ≤ 0.0001.

Since intranasal steroids induce T regulatory cells (31) and IL-3 was found associated with steroid treatment, we next wanted to investigate the role of recombinant IL-3 intranasal (i.n.) in a murine model of asthma. For this reason, we treated asthmatic WT mice in an OVA-induced asthma model with rIL-3 (**Figure 2A**). In this model, IL-3 was given i.n., during either the sensitization or the challenge phase of the disease to distinguish the effect of IL-3 on the immune system at different stages after antigen encounter. Here we found that, mice treated with rIL-3 during the sensitization or the challenge phase didn´t show a significant change in airway hyperresponsiveness as compared to OVA alone (**Supplementary Figure 1A**). In addition, mice treated with rIL-3 showed a trend to reduction of lung inflammation (**Supplementary Figures 2B, C**) and significant reduction of mucus production (**Figures 2B, C**) but no change in serum IgE levels (**Figure 2D**). Finally, in rIL-3-treated mice during the challenge phase, we found a downregulation of inflammatory type and induction of resident type eosinophils in the BAL fluid (BALF) of treated mice (**Figures 2E, F** and **Supplementary Figure 2C**).

### *In Vivo* Treatment With rIL-3 During the Allergen Challenge Phase and Decreased Lung GATA3+ ST2^int^ILC2 Cell Number

**Figure 3:**
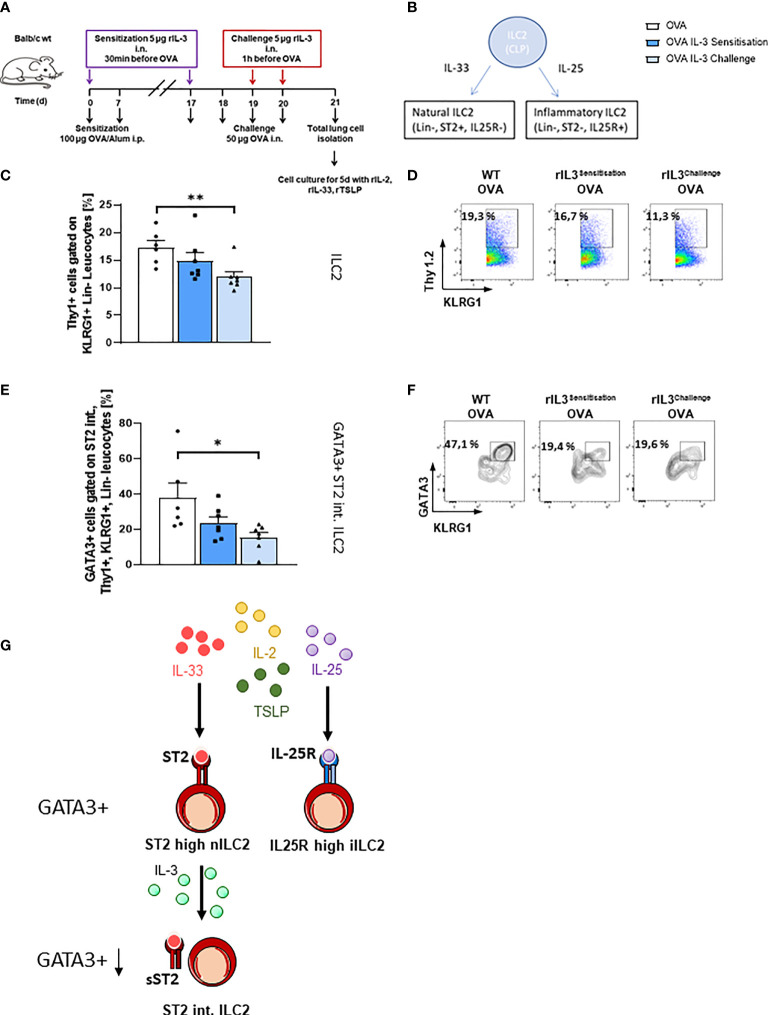
Lung ILC2s in an ovalbumin (OVA)-induced asthma model with intranasal rIL-3 application. **(A)** Experimental design for the induction of allergic asthma in wild-type (WT) mice treated with rIL-3 for the analysis of lung ILC2s. **(B)** Cartoon on the differentiation of different ILC2 subpopulations. **(C–F)** Flow cytometry analysis of ILC2 after *in vitro* differentiation. Flow cytometry analysis of lung ILC2s (%) **(C, D)**; flow cytometry analysis of GATA3+ ST2^int^. ILC2 **(E, F)** in the lung of WT mice with asthma and with and without additional rIL-3 treatment during sensitization or challenge phase (n = 6/7/7). **(G)** Cartoon on the mechanism of IL-3 involvement in ST2 shedding from ILC2. Data are presented as means ± SEMs. One-way ANOVA or Kruskal–Wallis test was used to calculate statistical significance. *p ≤ 0.05; **p ≤ 0.01.

We next asked if rIL-3 given during the allergen sensitization or challenge phase would influence the ILC2 phenotype. We further asked about the direct influence of rIL-3 on lung ILC2. To do so, we treated mice i.n. with rIL-3 during either the allergen sensitization or the allergen challenge phase. At the end of the asthma protocol on day 21, we isolated total lung cells and further differentiated them under ILC2 skewing conditions for 5 days (**Figure 3A**). As already described, there are two main subgroups of ILC2s, the natural (n) ILC2s and the inflammatory (i) ILC2s, which can be distinguished by the expression of ST2, which is a receptor subunit of IL-33 (32) (**Figure 3B**). Here, we found that rIL-3 treatment during the allergen sensitization or challenge phase resulted in the reduction of ILC2 cell number (**Figures 3C, D**). Finally, we found that GATA3+ST2^int^ ILC2 were significantly reduced in mice treated with rIL-3 *in vivo* during the challenge phase (**Figures 3E–G**), whereas the ST2^int^ ILC2 and the GATA3+ST2^high^ ILC2 were downregulated only by trend in mice treated *in vivo* with rIL-3 (**Supplementary Figures 1F–H**).

### Intranasal rIL-3 Induced IL-10-Producing ILC2 and Foxp3+ T Regulatory Cells in the Lung

**Figure 4:**
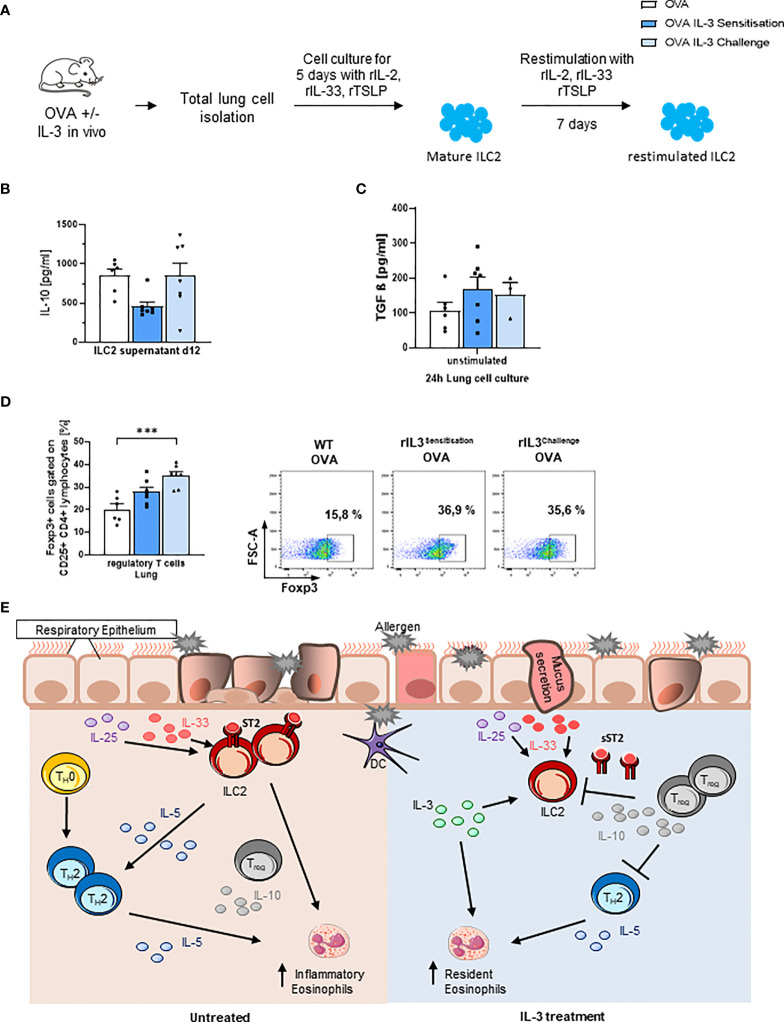
Intranasal rIL-3 application during challenge induces Treg cells. **(A)** Experimental design for lung ILC2 skewing. **(B)** After 5 days of *in vitro* ILC2 differentiation, IL-10 release was measured in the supernatants of ILC2 skewed from the lung of wild-type (WT) asthmatic mice with and without additional rIL-3 treatment during sensitization or challenge phase (n = 6/7/7). **(C)** TGF-beta level in 24-h unstimulated lung cell culture, measured by ELISA (n = 6/7/4). **(D)** Flow cytometry analysis of Foxp3+CD25+CD4+ regulatory T cells in total lung cells. A representative dot plot is shown for each group (n = 6/7/7). **(E)** Summary of the immunoregulatory role of IL-3 in allergic asthma. Data are presented as means ± SEMs. One-way ANOVA or Kruskal–Wallis test was used to calculate statistical significance. ***p ≤ 0.001.

Finally, we measured IL-10 in the supernatants of *in vitro* skewed ILC2s and found that ILC2s skewed *ex vivo* from mice that received IL-3 *in vivo* during the challenge phase released more IL-10 as compared to the those skewed from the lung cells of mice that received IL-3 during the sensitization phase *in vivo* (**Figures 4A, B** and **Supplementary Figures 1E**). We next looked for immunosuppressive molecules like active TGF-beta, which is known to induce Foxp3+ T regulatory cells, and we found that it was upregulated by trend in the lung of *in vivo* rIL-3-treated mice (**Figure 4C**).

Consistently, rIL-3 given during the challenge phase resulted in induction of CD4+CD25+Foxp3+ T regulatory cells in the lung of treated mice (**Figure 4D** and **Supplementary Figure 2B**).

## Discussion

In our human study, we describe an increased secretion of IL-3 in the upper airways of children with controlled asthma and PHA-stimulated PBMCs of asthmatic children with A-PC and specifically those children who were treated with steroids. These results, although restricted to a small cohort of children, challenged us to further investigate in detail why IL-3 is regulated under treatment conditions associated with the resolution of this worldwide increasing disease.

To check whether IL-3 is a negative regulator of lung inflammation, we correlated IL-3 production in the airways with the anti-inflammatory marker sST2 and found that IL-3 directly correlated with sST2 in the airways of asthmatic children treated with steroids. Moreover, we treated WT mice i.n. with rIL-3 in our murine model of asthma. Treating asthmatic mice with rIL-3 during the challenge phase reduced lung inflammatory eosinophils, mucus hyperproduction, and the number of ILC2s expressing GATA3 in the lung. The plasticity of ILC2 has been demonstrated in previous studies. Thus, the reduced GATA3+ILC2 found in the mice treated with IL-3 during the challenge phase might be the result of a shift into ILC1 in the presence of IL-12 or into ILC3 in the presence of TGF-β (33–35). However, this point has to be further investigated in the future.

The reduction of eosinophils during the challenge phase is consistent with the literature stating a downregulation of the chemokine receptor CCR3 on eosinophils. Furthermore, IL-3 was shown to negatively regulate the expression of IL-5Rα on eosinophils, resulting in reduced survival and migratory activity of eosinophils (36). In this model, we did not observe induction of basophils upon IL-3 treatment (**Supplementary Figure 1I**). These results are controversial to other studies describing increased numbers of basophils stimulated by IL-3 (37). Consistent with the decrease of inflammatory lung eosinophils, rIL-3 intranasal administration during the allergen challenge phase increased the release of the immunosuppressive cytokine TGF-beta, which along with IL-10 releasing ILC2 resulted in the induction of the immunosuppressive T regulatory cells. Therefore, treatment with rIL-3 during the challenge phase could offer a possible therapeutic application during seasonal allergen exposure. This is also consistent with the therapeutic use of inhaled steroids during exacerbations of disease.

IL-3 is discussed very controversially in the literature. In our model, we did not find beneficial effects of IL-3 application during the sensitization phase. This might also be due to the different application routes of OVA (i.p.) and rIL-3 (i.n.). In contrast, we see a protective immune regulatory response during the challenge phase, when rIL-3 was applied before challenging the mice i.n. with OVA. On the contrary, another OVA model showed reduced levels of eosinophils and bronchial hyperreactivity, when administering anti-IL-3 on the last day of the protocol, after the challenge (38). These findings imply that IL-3 might only be supportive during a certain time of the experimental model, in our case, before the challenge. It is also possible that IL-3 regulates different processes in a dose-dependent manner. Similar to our group, Srivastava et al. found increased numbers of Tregs upon application of increasing IL-3 doses. Furthermore, IL-3 pretreated Tregs were able to suppress T effector cell proliferation *in vitro* (39). The underlying mechanism might be that IL-3-dependent Treg induction leads to a suppression of ILC2 *via* immunosuppressive cytokine IL-10. This effect is only detectable when applying IL-3 prior to antigen challenge, as the Tregs will be boosted simultaneously with the activation of the inflammatory response, enabling them to control this immune reaction.

Another possibility is that IL-3 given during the challenge phase regulated locally the release of sST2 from the surface of ILC2 or other inflammatory cells expressing ST2. This possibility is also supported by our positive correlation between sST2 and IL-3 in the airways of asthmatic children treated with steroids. Consistent with this hypothesis is also the downregulation of the GATA-3 ST2 intermediate population after treatment with IL-3 during the challenge phase. This population might be the result of previous ST2 shedding of the ST2 high population (**Figure 3G**). Current literature shows that sST2 is able to suppress ILC2-derived IL-5 and IL-13 cytokine production upon IL-33 stimulation (40, 41). Thus, IL-3 might contribute to ST2 shedding in ILC2, which then becomes ST2 intermediate and cannot survive. Further experiments are needed in this direction.

Another interesting finding was on the natural helper cell population (NH cells), which belongs to the ILC family. These innate cells were found to be involved in steroid resistance and are able to produce large numbers of IL-5 and IL-13 upon activation (42). In the lung, these cells are stimulated by IL-33 and a cytokine milieu including IL-2, IL-7, and TSLP (43). IL-3 could be a possible regulator of these NH cells, interfering in their activation and production of TH2 cytokines.

However, in our experiment, we used ILC2s skewed from total lung cells, which are not freshly sorted ILC2s from the lung. Further investigation in this model is needed on the *in vivo* characteristics of this IL-3-responsive ILC2 population. Additional experiments in house dust mite-induced asthma models would also be of interest. Our human cohort has only a restricted number of participants, and a bigger cohort would certainly strengthen our findings. Further, analysis of IL-3Rα on Treg and ILC2 isolated from control and asthmatic subjects would extend our knowledge on the responsiveness of these cell types to IL-3.

In conclusion, we demonstrate here that IL-3 not only has pro-inflammatory properties but can also regulate the asthmatic burden *via* inducing T regulatory cell response and reducing ILC2 proliferation. Nevertheless, we are only at the early phase of understanding the possible dual role of rIL-3 for allergic asthma in development and therapy (**Figure 4E**).

## Data Availability Statement

The datasets generated for this study can be accessed upon request to the corresponding author.

## Ethics Statement

The studies involving human participants were reviewed and approved by the Ethics committee of the Friedrich-Alexander University Erlangen-Nürnberg. Written informed consent to participate in this study was provided by the participants’ legal guardian/next of kin. The animal study was reviewed and approved by the Government of Unterfranken, Bavaria.

## Author Contributions

The human study PreDicta was performed by previous members of the group at the Molecular Pneumology in Collaboration with the Children Hospital in Erlangen. SF supervised the entire work, analyzed the human data, and created **Figure 1**. SK and ZY performed the murine experiments, created the respective figures, and contributed with SF to the writing of the manuscript. TZ is the pediatrician who examined most of the children in PreDicta WP1‐UKER and made the medical diagnosis. NP designed the WP1 project PreDicta and was the coordinator of PreDicta. PX helped NP to set up the European PreDicta cohorts. CG coordinated the histology work presented in this manuscript. All authors contributed to the article and approved the submitted version.

## Funding

This work was supported by the European Grant PreDicta (Collaborative Project grant 260895) awarded to SF in Erlangen and in the other European Centers to NP in Athens. Furthermore, the work was supported by the Department of Molecular Pneumology and the SFB grant CRC1181/TP08 (Checkpoints for Resolution of Inflammation) awarded to SF. ZY is supported by the SFB grant CRC1181/TP08. SK is supported by an IZKF grant (Project A82) awarded to SF in Erlangen.

## Conflict of Interest

The authors declare that the research was conducted in the absence of any commercial or financial relationships that could be construed as a potential conflict of interest.

## Publisher’s Note

All claims expressed in this article are solely those of the authors and do not necessarily represent those of their affiliated organizations, or those of the publisher, the editors and the reviewers. Any product that may be evaluated in this article, or claim that may be made by its manufacturer, is not guaranteed or endorsed by the publisher.

## Abbreviations

AHR, airway hyperresponsiveness; BAL(F), bronchoalveolar lavage (fluid); Foxp3, Forkhead-box protein 3; GATA3, GATA-binding protein 3; (n/i) ILC2, (natural/inflammatory) type 2 innate lymphoid cells; i.n., intranasally; i.p., intraperitoneal; IgE, immunoglobulin E; IL, interleukin; IL3Rα, IL-3 receptor α; KLRG1, killer cell lectin-like receptor subfamily G member 1; Lin, lineage; OVA, ovalbumin; PAS, periodic acid-Schiff; PBMCs, peripheral blood mononuclear cells; Penh, enhanced pause; PHA, phytohemagglutinin; PreDicta, post-infectious immune reprogramming and its association with persistence and chronicity of respiratory allergic diseases; r, recombinant; TH, T helper cells; Treg, regulatory T cells; TSLP, thymic stromal lymphopoietin; unst, unstimulated; WT, wild type.
