# Supplementary material for: An Immunoregulatory Role of Interleukin-3 in Allergic Asthma
Source: Front Immunol. 2022 Feb 23;13:821658. doi: 10.3389/fimmu.2022.821658 (PMC8904351; doi:10.3389/fimmu.2022.821658)
Supplement: Supplementary file 1 [file DataSheet_1.docx]

Supplementary Material

An Immunoregulatory Role of Interleukin-3 in Allergic Asthma

Susanne Krammer^1,┼^, Zuqin Yang^1,┼^, Theodor Zimmermann^2^, Paraskevi Xepapadaki^3^, Carol I. Geppert^4^, Nikolaos G. Papadopoulos^3, 5^, and Susetta Finotto^1,┼ *^.

^1^ Department of Molecular Pneumology, Friedrich-Alexander-University (FAU) Erlangen-Nürnberg, Universitätsklinikum Erlangen, 91052 Erlangen, Germany

^2^ Children’s Hospital, Department of Allergy and Pneumology, Friedrich-Alexander- University (FAU) Erlangen-Nürnberg, Universitätsklinikum Erlangen, 91054 Erlangen, Germany

^3^ Allergy and Clinical Immunology Unit, 2nd Pediatric Clinic, National and Kapodistrian University of Athens, 11527 Athens, Greece

^4^ Institute of Pathology, Friedrich-Alexander- University (FAU) Erlangen-Nürnberg, Universitätsklinikum Erlangen, 91052 Erlangen, Germany

^5^ Centre for Respiratory Medicine & Allergy, Division of Infection, Immunity & Respiratory Medicine, University of Manchester, M13 9PL Manchester, United Kingdom of Great Britain and Northern Ireland

^┼^ These authors contributed equally to this manuscript and thus share the first authorship

*** Correspondence:**Prof. Dr. Dr. Susetta Finotto

Universitätsklinikum Erlangen

Abt. Molekulare Pneumologie

Hartmannstraße 14

91052 Erlangen

Phone: +49-9131-85-35883

[Mail: susetta.finotto@uk-erlangen.de](mailto:susetta.finotto@uk-erlangen.de)

[http://www.molekulare-pneumologie.uk-erlangen.de](http://www.molekulare-pneumologie.uk-erlangen.de/)

1. **Supplementary Tables**

## Supplementary Table 1. Demographic and clinical data of the healthy PreDicta cohort WP1-UK-ER analyzed at the baseline visit.

| **Control** | **Age** | **Gender** | **Skin Prick**  **Test*** | **Atopic dermatitis** | FEV1% predicted | CRP  (mg/ml) | IL3 [pg/ml] in Swab | PBMC SN PHA IL3 [pg/ml] |
| --- | --- | --- | --- | --- | --- | --- | --- | --- |
| C1 | 6 | male | n.d. | No | 77 | n.m. | 0.000 | n.m. |
| C2 | 6 | female | n.d. | No | 121 | 1,4 | 0.000 | 34,584 |
| C3 | 5 | male | n.d. | No | 110 | 0,29 | 7,356 | 86,646 |
| C4 | 4 | male | n.d. | No | 118 | 0,9 | 12,60 | 0,000 |
| C5 | 4 | female | n.d. | No | 111 | 0,68 | 9,88 | 0,000 |
| C6 | 5 | female | n.d. | No | n.e. | 0,48 | 8,17 | 0,000 |
| C7 | 5 | female | negative | No | 84 | 0,78 | 10,34 | 1,072 |
| C8 | 3 | male | n.d. | No | n.e. | 0,26 | 3,19 | 146,390 |
| C9 | 6 | male | n.d. | Yes | 105 | 0,22 | 12,88 | 8,290 |
| C10 | 4 | female | n.d. | No | 109 | 1,25 | 4,56 | 0,000 |
| C11 | 6 | male | n.d. | No | 87 | 21,9 | 7,17 | 0,000 |
| C12 | 4 | male | negative | No | 100 | 0,76 | 6,689 | 0,000 |
| C13 | 5 | female | n.d. | No | 112 | 0,79 | 25,24 | 0,000 |
| C14 | 5 | female | al | No | 119 | 1,74 | 0,000 | 0,000 |
| C15 | 4 | male | ca, f | No | 113 | n.m. | 0,58 | 63,964 |
| C16 | 5 | male | n.d. | No | 111 | 0,11 | n.m. | 0,000 |
| C17 | 4 | male | negative | No | 109 | 2,16 | n.m. | 0,000 |
| C18 | 4 | female | negative | No | 92 | 0,64 | n.m. | 0,000 |
| C19 | 5 | male | negative | No | 123 | 0,36 | n.m. | 12,434 |
| C20 | 4 | male | negative | No | 121 | 0,51 | n.m. | 0,000 |
| C21 | 5 | male | negative | Yes | 109 | 0,74 | n.m. | 0,000 |

*al, *Alternaria* species; ca, cat; f, *Dermatophagoides* mix; n.d., not done;n.m.=not measured.

## Supplementary Table S2: Clinical data of preschool children with uncontrolled (UC) and partially controlled (PC)

## asthma analyzed at the baseline visit in the PreDicta cohort WP1-UK-ER.

| Asthma Patient ID | Phenotype (PRACTALL 2008) | Asthma Control (GINA 2009) | FEV1% predicted | CRP  (mg/ml) | IL3 [pg/ml] in Swab |
| --- | --- | --- | --- | --- | --- |
| A16 | a, v | Uncontrolled | 92 | 0,5 | 15,292 |
| A17 | a, e, v | Uncontrolled | 111 | 1,01 | 11,298 |
| A43 | V | Uncontrolled | 69 | 2,90 | 0,00 |
| A2 | U | Partially Controlled | n. e. | n.m. | 0,000 |
| A3 | U | Partially Controlled | 95 | 1,22 | 0,000 |
| A5 | U | Partially Controlled | 102 | 2,13 | 0,000 |
| A7 | v, a | Partially controlled | 143 | 0,13 | 0,000 |
| A9 | v, a | Partially controlled | 115 | n.m. | 0,000 |
| A10 | v | Partially controlled | 98 | 5,34 | 0,000 |
| A13 | e | Partially controlled | 115 | 0,13 | 3,920 |
| A31 | v | Partially controlled | 71 | 1,87 | 4,356 |

Asthma Phenotype(PRACTALL 2008): v= virus-induced; a= allergen-induced e=exersice-induced; u=unresolved; *n.e.=not evaluable.*

## Supplementary Table 3. Antibodies used for flow cytometry analysis.

| **Antikörper** | **Fluorochrom** | **Klon** | **Hersteller** |
| --- | --- | --- | --- |
| Anti-Biotin | APC | Bio3-18E7 | Miltenyi Biotec GmbH, Bergisch Gladbach |
| CD3 | APC | 17A2 | eBioscience™, Thermo Fisher Scientific, Waltham, USA |
| CD4 | AlexaFluor647 | RM4-5 | BD Biosciences, Heidelberg |
| CD4 | PerCP-Cy5.5 | RM4-5 | BD Biosciences, Heidelberg |
| CD11b | V450 | M1/70 | BD Biosciences, Heidelberg |
| CD11c | APC | N418 | eBioscience™, Thermo Fisher Scientific, Waltham, USA |
| CD25 | BV421 | PC61 | BioLegend, San Diego, CA, USA |
| CD45.2 | FITC | 104 (RUO) | BD Biosciences, Heidelberg |
| CD49b | FITC | DX5 | BD Biosciences, Heidelberg |
| CD62L | PE-Cy5 | MEL-14 | Invitrogen, Thermo Fisher Scientific, Waltham, USA |
| CD90.2, Thy1.2 | PE-Cy7 | 53-2.1 | BD Biosciences, Heidelberg |
| CD101 | AlexaFluor647 | 307707 | BD Biosciences, Heidelberg |
| CD117, c-kit | APC | 2B8 | eBioscience™, Thermo Fisher Scientific, Waltham, USA |
| FcεRia | PE-Cy7 | MAR-1 | Invitrogen, Thermo Fisher Scientific, Waltham, USA |
| Foxp3 | AlexaFluor 647 | MF23 | BD Biosciences, Heidelberg |
| GATA3 | AlexaFluor 488 | L50-823 | BD Biosciences, Heidelberg |
| KLRG1 | V450 | 2F1 | BD Biosciences, Heidelberg |
| Siglec F | APC | E50-2440 | BD Biosciences, Heidelberg |
| Siglec F | BV510 | E50-2440 | BD Biosciences, Heidelberg |
| ST2, IL33R | BB700 | U29-93 | BD Biosciences, Heidelberg |

1. **Supplementary Figures**
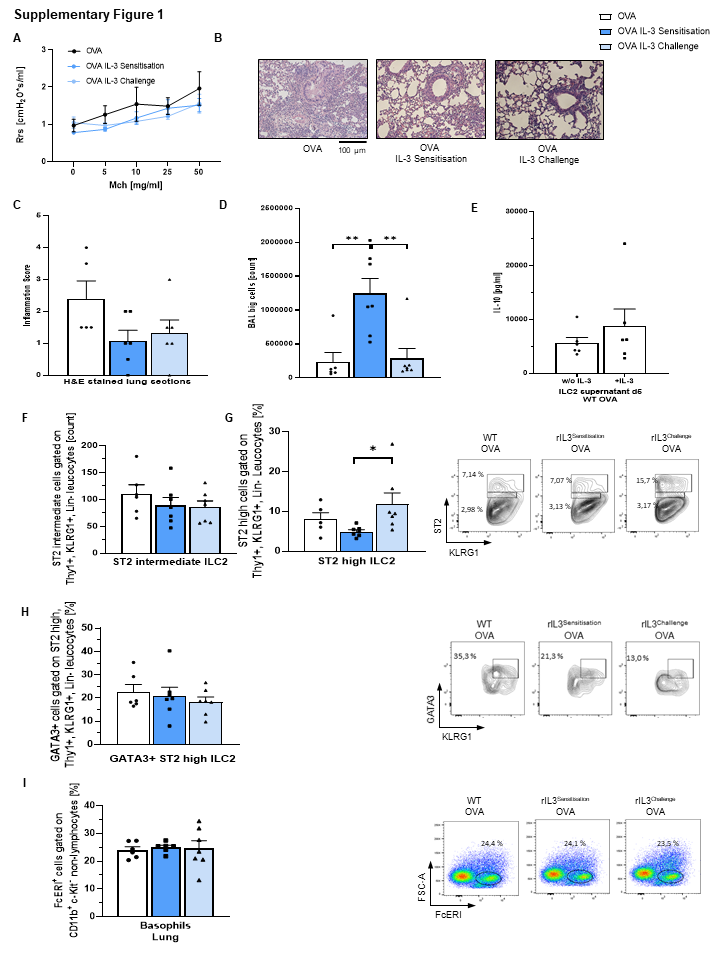


**Supplementary Figure 1.** **Intranasal rIL-3 application during sensitization or allergen challenge.** **(A)** Response to increasing doses of methacholine during invasive lung function measurement (Rrs) in OVA treated mice with and without rIL-3 in vivo. **(B, C)** Pathological score of the lung inflammation of mice with and without asthma (n=5/6/6). A representative light microscopic picture of the H&E staining of lung sections is shown for each group. **(D)** Cell count of big cells obtained by bronchoalveolar lavage. **(E)** IL-10 in the supernatant of ILC2 skewed for 12 days in present of IL-3 from OVA-induced asthmatic mice were quantified by ELISA (n=6/6) **(F)** Flow cytometry analysis of lung ILC2 ST2int. differentiated over 5 days (count). **(G).** Flow cytometry analysis of ST2hi GATA3+ KLRG1+Thy1+ ST2hi.Lin- ILC2s in the lung of WT asthmatic mice with and without additional rIL-3 treatment during sensitization or challenge phase after in vitro ILC2 skewing for 5 days (n=6/7/7). **(H)** Flow cytometry analysis of the percentage of basophils in total lung cells (n=6/7/7). A representative contour plot or dot plot is shown for each group. Data are presented as means ± SEMs. Two-way ANOVA was used to calculate statistical significance. * p ≤ 0.05; ** p ≤ 0.01.


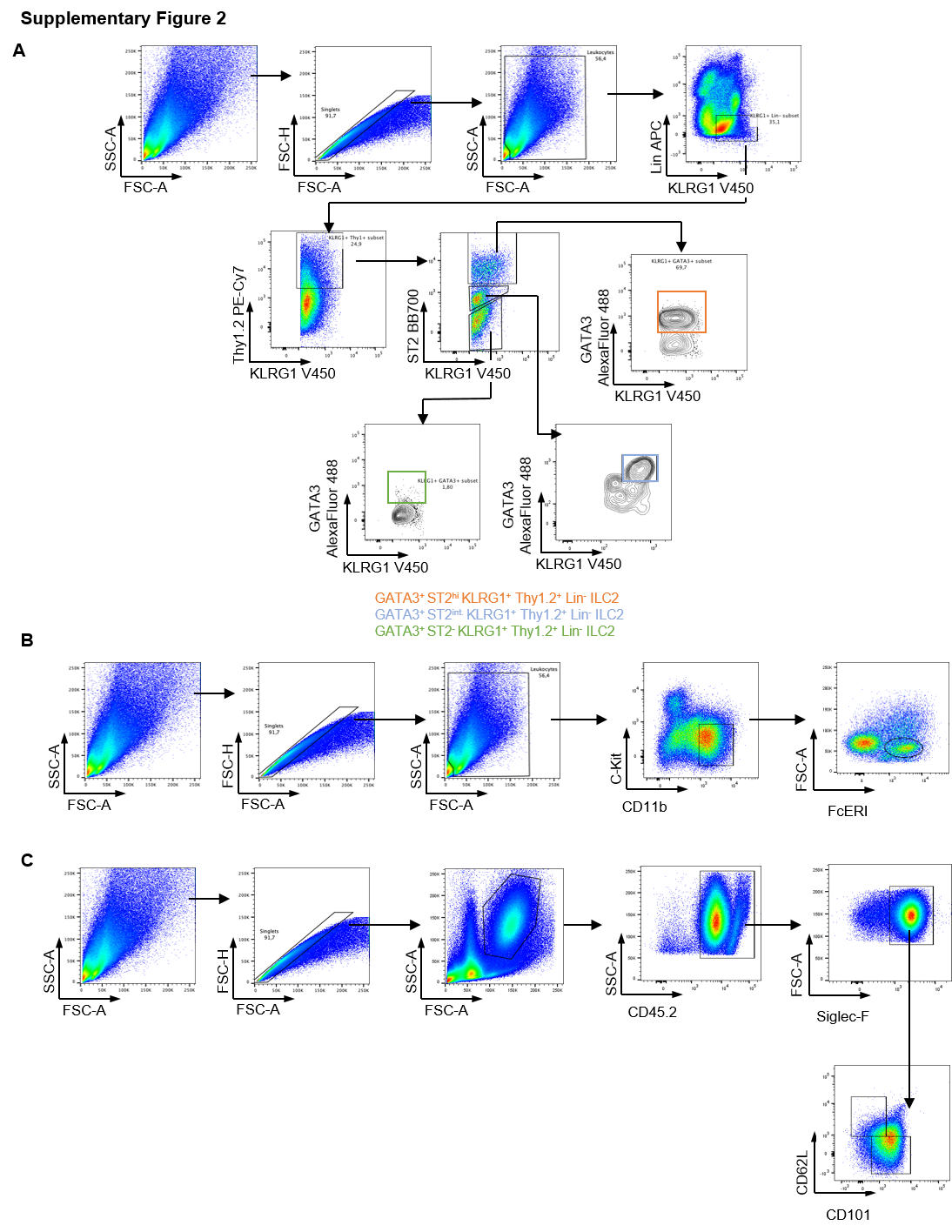


**Supplementary Figure 2. Flow cytometry analysis of lung ILC2s, basophils and eosinophils. (A)** Gating strategy for the flow cytometry analysis of ST2hi, ST2int. and ST2- KLRG1+ Thy1.2+ Lin- ILC2s from the lung of WT asthmatic mice with and without additional rIL-3 treatment after 5 days in culture. **(B)** Gating strategy for the flow cytometry analysis of basophils from isolated total lung cells. . **(C)** Gating strategy for the flow cytometry analysis of eosinophils from isolated lung cells.
